# Meibomian gland dysfunction in patients with thyroid-associated ophthalmopathy: a systematic review and meta-analysis

**DOI:** 10.3389/fmed.2025.1709057

**Published:** 2025-11-11

**Authors:** Yanling Lu, Yongran Li, Tulong Lin

**Affiliations:** 1 Guangzhou Panyu Bright Eye Hospital, Guangzhou, China; 2 Center of Ophthalmology, Heyou Hospital, Foshan, China; 3 Center of Ophthalmology, Zhongshan Torch Development Zone People's Hospital, Zhongshan, China

**Keywords:** thyroid-associated ophthalmopathy, meibomian gland dysfunction, dry eye, meibomian gland, meta-analysis

## Abstract

**Background:**

Meibomian gland dysfunction (MGD) secondary to thyroid-associated ophthalmopathy (TAO) represents a significant pathogenic mechanism in dry eye disease. This study provides the first systematic review and meta-analysis of MGD indicators in TAO.

**Methods:**

The study protocol was prospectively registered with the International Prospective Register of Systematic Reviews (PROSPERO) (Registration ID: CRD420251020327) before data extraction. Following PRISMA and MOOSE guidelines, a systematic search was conducted across PubMed, Embase, Web of Science, Scopus, Ovid Medline, and Cochrane from inception through March 27, 2025. Fourteen studies met the inclusion criteria. Key indicators included lipid layer thickness (LLT), meiboscore, meibum quality, first non-invasive tear film break-up time (NITBUT-f), average non-invasive tear film break-up time (NITBUT-avg), tear break-up time (TBUT), meibomian gland dropout area in the upper (MGDU) and lower eyelids (MGDL), and *in vivo* confocal microscopy (IVCM) markers (meibomian gland acinar density [MAD], meibomian gland acinar longest diameter [MALD], meibomian gland acinar shortest diameter [MASD]). Risk of bias was assessed using the AHRQ checklist or NOS. Meta-analysis was performed with Review Manager 5.4.1 and Stata 16.0. Publication bias was assessed using Egger's test and funnel plots. Fixed-effects models were used in the absence of significant heterogeneity (*P* > 0.10 or *I*^2^ < 50%); otherwise, random-effects models were applied.

**Results:**

Thirteen studies (813 TAO eyes, 522 controls) were included in the meta-analysis. Quality assessment revealed moderate-to-high methodological rigor across studies. Patients with TAO exhibited significantly worse meibomian gland indicators compared to controls: shorter tear film stability (NITBUT-f, TBUT), higher LLT, increased meiboscore and greater eyelid gland dropout (MGDU, MGDL). IVCM markers indicated meibomian acinar enlargement (MALD, MASD). Significant heterogeneity was observed in several outcomes, including NITBUT-f, NITBUT-avg, meiboscore, Meibum quality, TBUT and MGDU comparisons.

**Conclusions:**

Despite the limited number of studies and small sample sizes, TAO is linked to meibomian gland atrophy, acinar dilation, and tear film instability. Active disease is associated with more pronounced lipid layer abnormalities. Targeted evaluation and management of MGD are crucial to mitigate TAO-associated ocular surface morbidity and improve patient quality of life.

## 1 Background

Thyroid-associated ophthalmopathy (TAO) is a chronic inflammatory orbital disorder strongly linked to autoimmune thyroid diseases, driven by cross-reactive immune responses targeting shared antigens, such as the thyrotropin receptor (TSHR) (1, 2). Pathologically, TAO is characterized by orbital fibroblast proliferation, glycosaminoglycan deposition, and adipose tissue hyperplasia. Clinically, it manifests as ocular surface damage, progressive proptosis, eyelid retraction, extraocular muscle hypertrophy, and diplopia. Severe cases may lead to vision-threatening complications, including compressive optic neuropathy and corneal ulceration (3, 4).

Ocular surface dysfunction, common in patients with TAO, is primarily manifested as conjunctival hyperemia, dry eye disease (DED), and exposure keratitis, significantly impairing quality of life (5, 6). The pathogenesis of TAO-associated dry eye is multifactorial: autoantibodies target TSHR-expressing lacrimal glands, inducing inflammatory edema and fibrosis that disrupt reflex tear secretion (7, 8). Elevated tear inflammatory cytokines (e.g., IL-1β, IL-6, IL-8) further exacerbate ocular surface inflammation, damaging conjunctival goblet cells and accessory lacrimal glands, thus reducing mucin production and basal tear secretion (9, 10). Mechanical factors, such as proptosis-induced lagophthalmos, increase corneal exposure, while fibrotic levator palpebrae complexes contribute to incomplete blinking, accelerating tear evaporation (11). Long-term immunosuppression or radiotherapy in moderate-to-severe TAO may further compromise the epithelial barrier (12). Conventional artificial tears have limited efficacy (< 50%) in managing TAO-associated DED, highlighting the urgent need for targeted therapeutic approaches (13).

Meibomian gland dysfunction (MGD), a major cause of evaporative DED, has gained increasing recognition for its role in the pathogenesis of TAO-associated ocular surface dysfunction (14). Patients with TAO exhibit significant meibomian gland dropout, particularly in the upper eyelids, with markedly reduced acinar density and gland orifice size compared to healthy controls. This leads to insufficient lipid secretion, decreased tear film lipid layer thickness, and accelerated tear evaporation (15, 16). Elevated systemic and local proinflammatory cytokines (e.g., IL-1β, TNF-α) impair the regenerative capacity of meibomian glands and exacerbate lipid layer instability. Concurrent corneal subbasal nerve damage reduces blink frequency and disrupts neurotrophic signaling, further worsening ductal keratinization and abnormal lipid composition (17). Therefore, TAO-associated MGD necessitates further investigation into its mechanisms and potential therapeutic strategies.

Recent studies have increasingly elucidated the clinical significance, epidemiological trends, and pathophysiological mechanisms of TAO-associated MGD. To our knowledge, no systematic review has comprehensively evaluated the scope of MGD in TAO. This meta-analysis synthesizes global evidence by integrating data from multinational cohort studies, aiming to clarify the clinical manifestations, pathophysiological pathways, and correlations with disease activity in TAO-associated MGD. The findings will provide a theoretical foundation for optimizing therapeutic strategies targeting MGD.

## 2 Methods

### 2.1 Search strategy for study selection

This systematic review adhered to the Preferred Reporting Items for Systematic Reviews and Meta-Analyses (PRISMA) guidelines and the Meta-analysis of Observational Studies in Epidemiology (MOOSE) checklist (Supplementary Files 1 and 2) (18, 19). The study protocol was prospectively registered with the International Prospective Register of Systematic Reviews (PROSPERO; Registration ID: CRD420251020327). Two independent investigators (L.Y.L. and L.Y.R.) conducted a comprehensive search of six databases (PubMed, Embase, Web of Science, Scopus, Ovid Medline, and Cochrane Library) from inception through March 27, 2025. To capture critical emerging evidence, studies published after the search cutoff date but prior to manuscript submission were included provided they met prespecified eligibility criteria. The search used a combination of Medical Subject Headings (MeSH) terms and free-text keywords: (“meibomian glands” OR “meibomian gland dysfunction” OR “MGD”) AND (“graves ophthalmopathy” OR “thyroid eye disease” OR “thyroid-associated ophthalmopathy” OR “thyroid-associated orbitopathy” OR “graves orbitopathy” OR “graves eye disease”). Additional studies were identified through manual searches of reference lists, with full search strategies provided in the Supplementary File 3.

Eligibility criteria were defined according to the PICOS framework (20): Population – patients diagnosed with TAO based on Bartley's criteria, European Group on Graves' Orbitopathy (EUGOGO) guidelines, and the American Thyroid Association/European Thyroid Association (ATA/ETA) consensus (21, 22); Intervention – not applicable; Comparison – TAO vs. healthy controls, and active vs. inactive TAO subgroups; Outcome – meibomian gland functional indicators; Study design – English-language cross-sectional or longitudinal studies. Exclusion criteria included: (1) animal studies; (2) secondary literature (e.g., reviews, case reports); (3) studies lacking primary data; (4) non-English publications.

Citavi v5.3 (Swiss Academic Software) was utilized for reference management and duplicate removal. The screening process was carried out in two stages by two independent reviewers (L.Y.L. and L.Y.R.): (1) title/abstract screening; (2) full-text assessment against the inclusion/exclusion criteria. Discrepancies were resolved through discussion with a third investigator (L.T.L).

### 2.2 Data extraction

Data extraction was conducted using a predefined template that captured details such as the first author, publication year, country, ethnicity, sex, age, Clinical Activity Score (CAS), devices, study design, and meibomian gland functional indicators. Indicators eligible for meta-analysis included: lipid layer thickness (LLT), meiboscore, meibum quality, first non-invasive tear film break-up time (NITBUT-f), average non-invasive tear film break-up time (NITBUT-avg), tear break-up time (TBUT), meibomian gland dropout area of the upper (MGDU) and lower eyelids (MGDL), and *in vivo* confocal microscopy (IVCM) indicators (meibomian gland acinar density [MAD], meibomian gland acinar longest diameter [MALD], meibomian gland acinar shortest diameter [MASD]). According to the EUGOGO guideline (22), a CAS ≥3/7 was defined as active TAO, while CAS ≤ 2/7 indicated inactive TAO. All definitions adhered strictly to the standardized descriptions from the original studies.

Meiboscore quantified total gland loss across the upper and lower eyelids using a 0–3 grading scale for each eyelid: 0 (no loss), 1 (< 1/3 area loss), 2 (1/3–2/3 loss), and 3 (> 2/3 loss). The composite score per eye (range 0–6) was the sum of the upper and lower eyelid grades. Meibum quality was graded on a 0–3 scale: 0 (clear fluid), 1 (cloudy fluid), 2 (cloudy particulate fluid), and 3 (toothpaste-like or non-expressible) (23). LLT, NITBUT-f, and NITBUT-avg were measured objectively via non-invasive ocular surface analyzers. LLT was defined as the vertical optical thickness of the tear lipid layer, with only mean values included. NITBUT-f represented the time from the first complete blink to the initial dry spot detection, while NITBUT-avg denoted the mean of regional first-breakup times.

The TBUT test was performed by applying a fluorescein strip to the conjunctival sac. After natural blinking, the time between the last complete blink and the first corneal dry spot, observed under cobalt blue slit-lamp illumination, was recorded.

MGDU and MGDL were quantified based on infrared meibography images obtained from an automated ocular surface analyzer. The percentage of meibomian gland dropout area in the upper and lower eyelids was calculated through either manual measurement or automated analysis.

IVCM assessments (HRT III Corneal Rostock Module, Heidelberg Engineering, Germany) focused on the lower eyelids, with masked operators capturing acinar images in standardized 400 × 400 μm fields. Two independent masked evaluators selected three high-quality, non-overlapping images from the nasal, central, and temporal regions of each lower eyelid (nine images per eyelid) for quantitative analysis. Indicators included in the meta-analysis were MAD, MALD, and MASD. MAD was calculated by manually counting the number of acinar units per image using ImageJ software (National Institutes of Health, Bethesda, MD) and converting the count to acinar units per mm^2^. MALD and MASD were measured manually using ImageJ software's linear tool, determining the longest and shortest axes of each acinar unit and averaging all analyzed values.

Two investigators (L.Y.L. and L.Y.R.) independently performed blinded data extraction. Discrepancies were resolved through consensus with a third reviewer (L.T.L.).

### 2.3 Risk of bias assessment

Cross-sectional studies were evaluated using the US Agency for Healthcare Research and Quality (AHRQ) checklist (11 items), which covers data sources, variable definitions, and sample representativeness (24–26). The methodological quality of case-control studies was assessed using the Newcastle-Ottawa Scale (NOS), which evaluates three domains: participant selection, group comparability, and exposure/outcome ascertainment, with a maximum score of 9. Two independent investigators (L.Y.L. and L.Y.R.) conducted the risk-of-bias assessments, resolving discrepancies through discussion with a third reviewer (L.T.L.). Studies scoring < 6 on the AHRQ checklist or < 5 on the NOS were classified as low quality with a high risk of bias and excluded from the meta-analysis to ensure robustness.

### 2.4 Statistical analysis

Statistical analyses were conducted using Review Manager (RevMan) v5.4.1 (Cochrane Collaboration) and Stata v16.0 (Stata Corp). Comparisons included: (1) patients with TAO vs. healthy controls; (2) active vs. inactive TAO subgroups. Continuous outcomes were expressed as mean ± standard deviation. For studies reporting medians with interquartile ranges, skewed data were first transformed using the Quantile estimation method by McGrath et al., followed by estimation of mean and SD using the methods proposed by Luo et al. and Wan et al. (27–29). Studies stratifying TAO into active/inactive subgroups were pooled using Cochrane-recommended formulas to derive combined group means and standard deviations (30).

Mean differences (MD) with 95% confidence intervals (CI) were calculated for outcomes with consistent units across studies. Heterogeneity was assessed using χ^2^ tests and I^2^ statistics: fixed-effect models were applied when *P* > 0.10 and *I*^2^ < 50%; random-effects models were used otherwise. Sensitivity analyses (leave-one-out method) were performed for outcomes with ≥ 3 studies. Publication bias was assessed using funnel plots for all outcomes; for analyses with ≥10 studies, Egger's test was additionally performed. Statistical significance was set at *P* < 0.05.

## 3 Results

### 3.1 Literature search results

**Figure 1:**
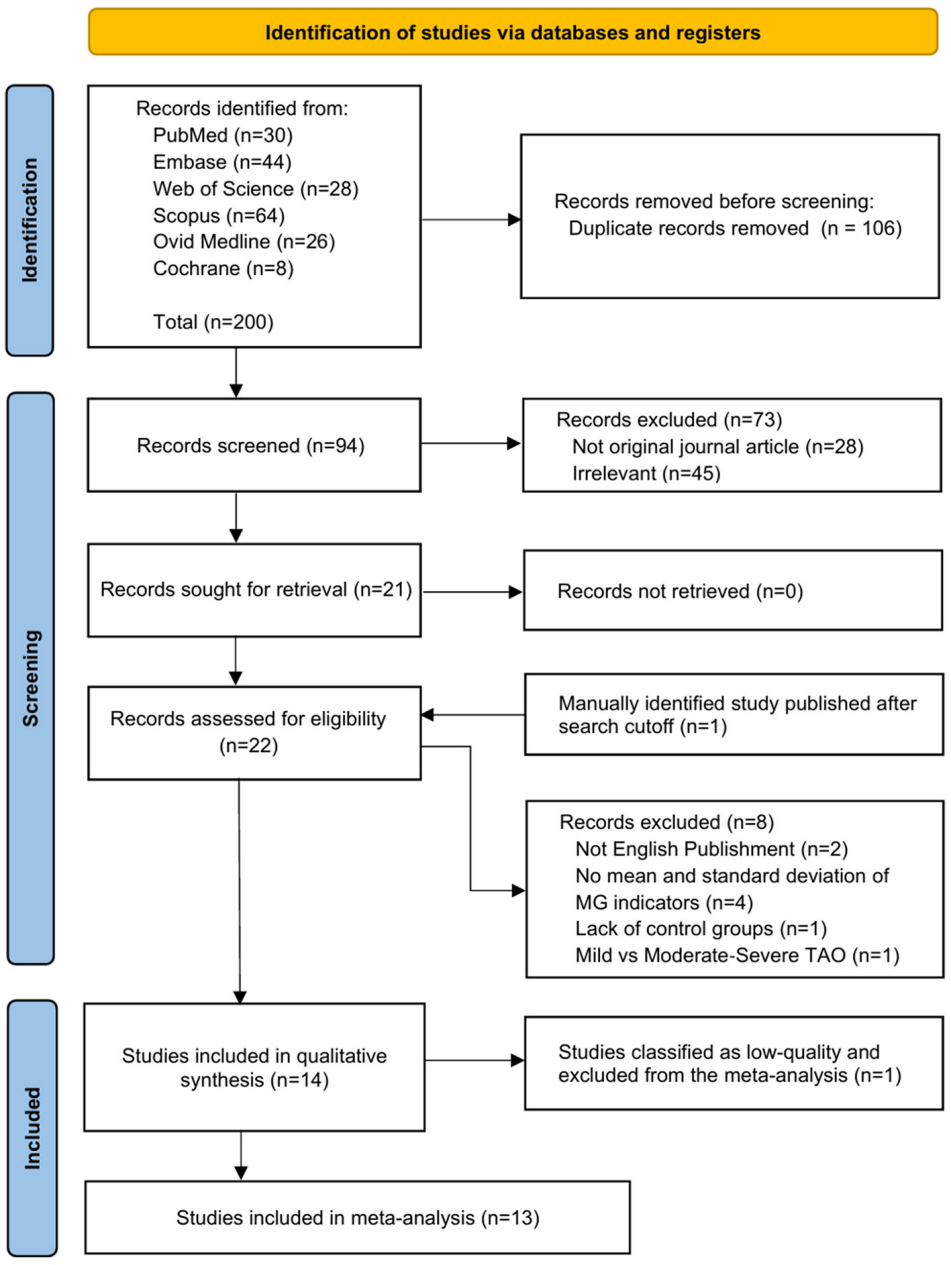
PRISMA flowchart of selection of studies for inclusion in the meta-analysis.

The database search initially identified 200 articles. After removing 106 duplicates, titles, abstracts, and article types of the remaining 94 articles were screened. A total of 73 articles were excluded due to irrelevance to the topic (*n* = 45) or ineligible publication types (*n* = 28). Full-text assessments of the remaining 21 articles led to the exclusion of 2 non-English publications, 4 articles that did not report mean values and standard deviations for meibomian gland indicators, 1 article lacking a control group, and 1 article missing subgroups based on eligibility criteria. Additionally, 1 article manually identified after the search date cutoff was incorporated. Consequently, 14 articles were included in the qualitative synthesis. One article was excluded due to low quality based on the quality assessment, leaving 13 articles included in the meta-analysis. The selection process adhered to PRISMA standards, as illustrated in the flow diagram (Figure 1).

### 3.2 Primary outcomes and qualitative synthesis

This systematic review included 14 studies (Table 1) published between 2015 and 2025, comprising 4 studies from China (31–34), 3 from Turkey (35–37), 2 from South Korea (38, 39)and 1 each from Brazil (40), Egypt (41), Thailand (42)], Italy (43), and Japan (44), with participants of Asian, Caucasian, and mixed ethnicities. The studies included 8 cross-sectional comparative and 6 retrospective case-control designs, involving 813 eyes with TAO and 522 healthy control eyes. Case definitions included active/inactive TAO and euthyroid TAO. The mean age of patients ranged from 38.5 to 57.3 years across the 14 studies, with one study reporting a median age of 40.00 years (36). Sex distribution was reported in 13 studies (301 eyes from males, 686 from females) (31–33, 35–44). CAS data were available in 11 studies (31–36, 38–41, 43), with active TAO subgroups showing mean CAS scores of 3.55–3.76, while non-stratified or inactive groups ranged from 0.79 to 3.1, with 1 study reporting a median CAS of 0.5.

**Table 1:** Characteristics of included studies.

**Studies/year**	**Country**	**Ethnicity**	**Definition of cases**	**Number of eyes**	**Gender (M/F)**		**Age (Mean** ±**SD) or (Median [IQR])**		**CAS (Mean ±SD) or (Median [IQR])**	**Devices**	**MG Indicators**
					**Cases**	**Controls**	**Cases**	**Controls**			
Guleser 2025 (35)	Turkey	Caucasian	Inactive TAO	TAO 33 Controls 36	7/26	8/28	42.79 ± 12.29	43.14 ± 12.01	0.79 ± 0.92	Sirius system	NITBUT-f, NITBUT-avg
Lai 2024 (31)	China	Asian	Euthyroid TAO	TAO 34 Controls 34	17/17	17/17	57.3 ± 13.9	57.3 ± 11.1	1.1 ± 1.1	Lipiview, OCULUS keratograph 5M	LLT, Meibum quality, NITBUT-avg, MGDU, MGDL
Liao 2023 (32)	China	Asian	TAO	TAO 152 Controls 93	12/64	10/51	42.99 ± 12.28	43.52 ± 17.93	0.88 ± 1.19	Lipiview, OCULUS keratograph 5M	LLT, NITBUT-f, NITBUT-avg
Riguetto 2023 (40)	Brazil	Mixed	Active, inactive TAO	Active 17 Inactive 16 Controls 18	Active 5/12 Inactive 3/13	4/14	Active 53.00 ± 14.34 Inactive 50.13 ± 15.44	49.63 ± 12.48	Active 3.76 ± 1.09 Inactive 0.88 ± 0.81	OCULUS keratograph 5M	NITBUT-f, NITBUT-avg, TBUT
Yilmaz 2023 (36)	Turkey	Caucasian	Inactive TAO	TAO 52 Controls 32	26/26	14/18	40.00 (31.00-50.50)	35.00(24.75–54.25)	0.50 (0.00–1.00)	Sirius system	Meiboscore, Meibum quality, NITBUT-f, NITBUT-avg, MGDU, MGDL
Yilmaz 2022 (37)	Turkey	Caucasian	Inactive TAO	TAO 44 Controls 38	20/24	18/20	43.6 ± 2.4	43.3 ± 2.2	NA	Sirius system	Meiboscore, TBUT
Allam 2021 (41)	Egypt	Caucasian	Active and inactive TAO	Active 20 Inactive 20 Controls 20	Active 7/13 Inactive 4/16	8/12	Active 40.75 ± 10.33 Inactive 38.50 ± 9.01	45.55 ± 7.16	Active 3.55 ± 0.60 Inactive 1.40 ± 0.50	IDRA device	Not included in the meta-analysis
Cheng 2021 (33)	China	Asian	Active and inactive TAO	Active 34 Inactive 46 Controls 62	9/31	10/21	Active 50.65 ± 9.49 Inactive 46.50 ± 10.80	45.65 ± 14.63	Active 3.56 ± 0.69 Inactive 0.96 ± 0.72	Lipiview, Sirius system, HRT III Corneal Rostock Module	LLT, Meibum quality, NITBUT-f, NITBUT-avg, MAD, MALD, MASD
Satitpitakul 2021 (42)	Thailand	Asian	Inactive TAO	TAO 106 Controls 106	16/37	16/37	51.2 ± 14.6	51.3 ± 15.8	NA	Lipiview, OCULUS keratograph 5M	LLT, Meibum quality, TBUT, MGDU
Vagge 2021 (43)	Italy	Caucasian	TAO	TAO 21 Controls 24	3/18	4/20	44.2 ± 9.9	39.8 ± 10.7	3.1 ± 2.1	HRT III Corneal Rostock Module	TBUT, MAD, MALD, MASD
Inoue 2020 (44)	Japan	Asian	TAO	TAO 38 Controls 14	2/17	0/14	44.0 ± 10.0	44.6 ± 7.6	NA	NA	Meiboscore, Meibum quality, TBUT
Park 2019 (38)	Korea	Asian	Active and inactive TAO	Active 20 Inactive 78	30/68	NA	Active 50.9 ± 8.8 Inactive 44.2 ± 12.8	NA	Total 1.9 ± 1.5	Lipiview	LLT, TBUT
Wang 2018 (34)	China	Asian	TAO	TAO 31 Controls 31	NA	NA	44.7 ± 11.0	44.7 ± 11.2	Total 1.6 ± 0.7	Lipiview II	LLT
Kim 2015 (39)	Korea	Asian	TAO	TAO 51 Controls 14	17/34	14/17	42.35 ± 12.80	45.45 ± 16.73	2.31 ± 1.59	NA	Meiboscore, TBUT

Lipiview and Lipiview II Ocular Surface Interferometer (TearScience, Inc., USA), OCULUS keratograph 5M (Oculus Optikgeräte GmbH, Germany), Sirius system (CSO, Italian), IDRA device (SBM Sistemi, Italy), HRT III Corneal Rostock Module (Heidelberg Engineering GmbH, Germany), LLT lipid layer thickness, NITBUT-f first non-invasive tear film break-up time, NITBUT-avg average non-invasive tear film break-up time, TBUT tear break-up time, MGDU meibomian gland dropout area of upper eyelid, MGDL meibomian gland dropout area of lower eyelid, MAD meibomian gland acinar density, MALD meibomian gland acinar longest diameter, MASD meibomian gland acinar shortest diameter.

Six studies reported LLT (31–34, 38, 42), four assessed meiboscore (36, 37, 39, 44), and five evaluated meibum quality (31, 33, 36, 42, 44). Tear film stability indicators included NITBUT-f (five studies) (32, 33, 35, 36, 40), NITBUT-avg (six studies) (31–33, 35, 36, 40), and TBUT (seven studies) (37–40, 42–44). MGDU and MGDL were quantified in two studies (31, 36), while two studies employed IVCM to assess MAD, MALD, and MASD (33, 43). Subjective indicators included meibum quality, TBUT, and IVCM-derived metrics (MAD, MALD, MASD), with standardized definitions and protocols for meibum quality and TBUT across all studies. Both IVCM studies utilized the HRT III Corneal Rostock Module (Heidelberg Engineering GmbH, Germany) for lower eyelid imaging, with masked analysis. Vagge et al. (43) specified imaging at 20–70 μm subepithelial depth, while Cheng et al. (33) did not report the exact depth.

Objective metrics included LLT, meiboscore, NITBUT-f, and NITBUT-avg. LLT measurements were consistently obtained using Lipiview and Lipiview II Ocular Surface Interferometers (TearScience, Inc., USA) across all six studies (31–34, 38, 42). For NITBUT assessments, four studies used the OCULUS Keratograph 5M (31, 32, 40, 42) (Oculus Optikgeräte GmbH, Germany) and the Sirius system (33, 35–37) (CSO, Italy), while one study employed the IDRA device (41) (SBM Sistemi, Italy). MGDU and MGDL were semi-objectively assessed: Lai et al. (31) used infrared meibography with the OCULUS Keratograph 5M (Oculus Optikgeräte GmbH, Germany) and manually quantified dropout areas via ImageJ software, while Yilmaz et al. (36) employed the Sirius system (CSO, Italy) for automated dropout area analysis.

### 3.3 Quality assessment

All included cross-sectional studies scored between 6 and 8 points on the AHRQ scale, indicating moderate to high methodological quality. The case-control studies scored between 4 and 7 points on the NOS. One study (41), scoring 4, was rated as low quality due to deficiencies in case selection and comparability and was excluded from the meta-analysis. The remaining studies demonstrated moderate to high quality (Tables 2, 3).

**Table 2:** Risk of bias assessment of included cross-sectional studies.

**Studies**	**1**	**2**	**3**	**4**	**5**	**6**	**7**	**8**	**9**	**10**	**11**	**Score**
Guleser 2025 (35)	Y	Y	N	Y	Y	Y	Y	N	U	Y	U	7
Lai 2024 (31)	Y	N	Y	U	Y	Y	Y	Y	U	Y	U	7
Liao 2023 (32)	Y	Y	Y	U	N	N	Y	Y	U	Y	U	6
Rigurtto 2023 (40)	Y	Y	Y	Y	N	N	Y	Y	U	Y	U	7
Cheng 2021 (33)	Y	Y	Y	Y	Y	Y	Y	N	U	Y	U	8
Satitpitakul 2021 (42)	Y	Y	Y	U	Y	Y	Y	N	U	Y	U	7
Vagge 2021 (43)	Y	Y	Y	Y	Y	Y	Y	N	U	Y	U	8
Park 2019 (38)	Y	Y	Y	Y	N	N	Y	N	U	Y	U	6

Y yes, N no, U unclear. US Agency for Healthcare Research and Quality, AHRQ: 1 = Define the source of information (survey, record review); 2 = List inclusion and exclusion criteria for exposed and unexposed subjects (cases and controls) or refer to previous publications; 3 = Indicate time period used for identifying patients; 4 = Indicate whether or not subjects were consecutive if not population-based; 5 = Indicate if evaluators of subjective components of study were masked to other aspects of the status of the participants; 6 = Describe any assessments undertaken for quality assurance purposes (e.g., test/retest of primary outcome measurements); 7 = Explain any patient exclusions from analysis; 8 = Describe how confounding was assessed and/or controlled; 9 = If applicable, explain how missing data were handled in the analysis; 10 = Summarize patient response rates and completeness of data collection; 11 = Clarify what follow-up, if any, was expected and the percentage of patients for which incomplete data or follow-up was obtained.

**Table 3:** Risk of bias assessment of included case-control studies.

**Studies**	**Selection**				**Comparability**	**Outcome**			**Score**
	**1**	**2**	**3**	**4**	**5**	**6**	**7**	**8**	
Yilmaz 2023 (36)	1	1	0	1	1	1	1	1	7
Yilmaz 2022 (37)	1	1	0	1	1	0	1	1	7
Allam 2021 (41)	1	0	0	1	0	0	1	1	4
Inoue 2020 (44)	1	0	0	1	1	0	1	1	5
Wang 2018 (34)	1	1	1	1	1	0	1	1	7
Kim 2015 (39)	1	1	1	1	1	0	1	1	7

The Newcastle–Ottawa Scale for case-control studies, NOS: 1 = Is the case definition adequate; 2 = Representativeness of the cases; 3 = Selection of Controls; 4 = Definition of Controls; 5 = Comparability of cases and controls on the basis of the design or analysis; 6 = Ascertainment of exposure; 7 = Same method of ascertainment for cases and controls; 8 = Non-Response rate.

Publication bias was evaluated using funnel plots across all 11 meta-analyses, each containing fewer than 10 included studies. The assessment identified minimal publication bias for four specific outcomes: LLT and MGDL between TAO and controls, as well as LLT and TBUT between active TAO and inactive TAO. The studies included in four outcomes were symmetrical scatter distribution alongside the axis, indicating minimal publication bias. The remaining seven analyses employing random-effects models, where auxiliary lines were not generated. Nevertheless, studies distributed evenly and symmetrically on both sides of the axis, suggesting possible low-level publication bias (Supplementary File 4).

### 3.4 Results of meta-analysis

#### 3.4.1 Meibomian gland indicators of patients with TAO vs. controls

**Figure 2:**
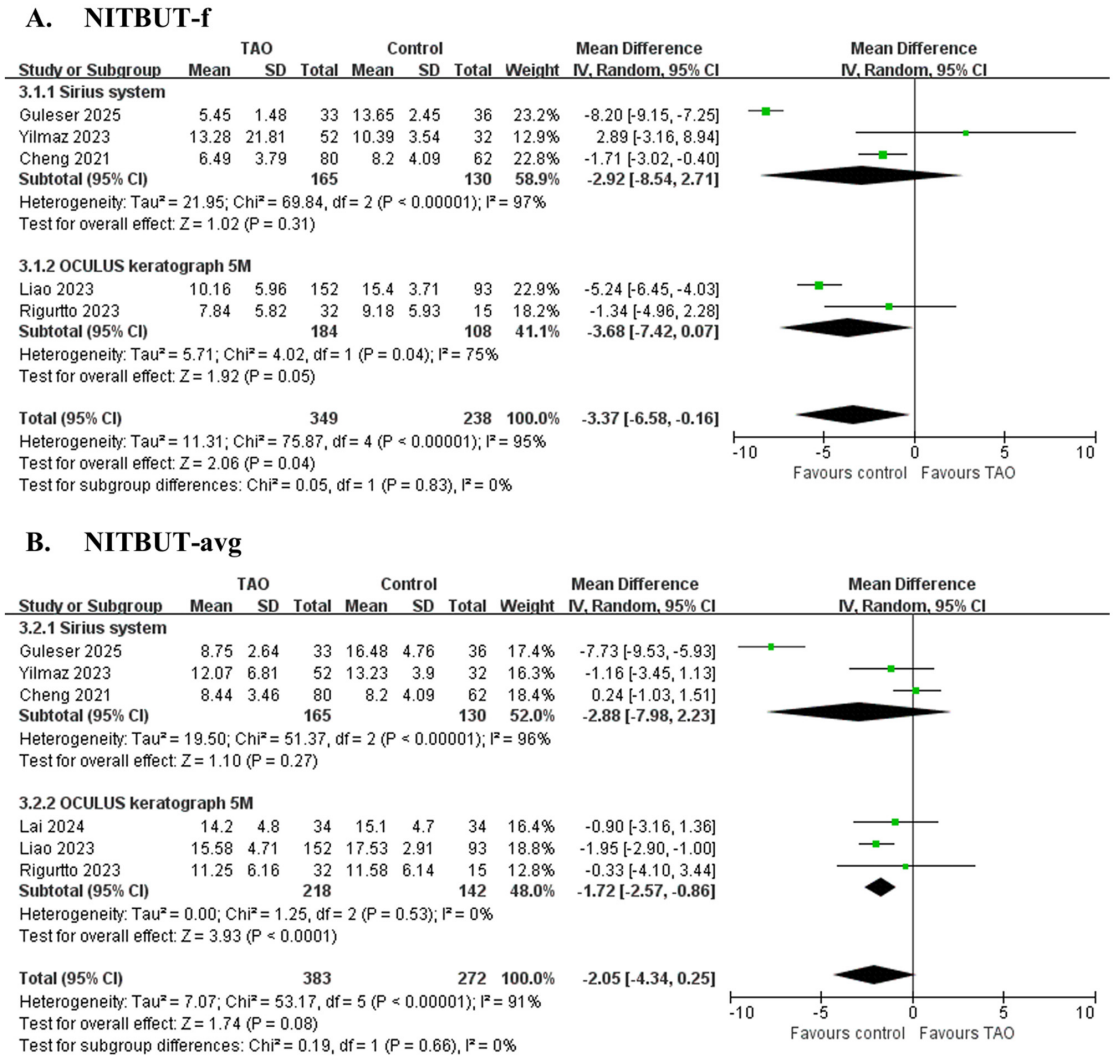
Forest plot of pooled difference in first non-invasive tear film break-up time (NITBUT-f), average non-invasive tear film break-up time (NITBUT-avg) between TAO and controls. **(A)** NITBUT-f; **(B)** NITBUT-avg.

Five studies (32, 33, 35, 36, 40) involving 587 eyes (Figure 2A and Table 4) showed no significant difference in NITBUT-f between patients with TAO and controls (MD = −3.37 s, 95% CI: −6.58 to −0.16; *P* = 0.04), with significant heterogeneity (*I*^2^ = 95%, *P* < 0.001). Subgroup analysis suggests that heterogeneity is not associated with device type. Sensitivity analysis revealed critical dependence on individual studies: exclusion of Guleser et al. (35) altered the MD to −2.17 s (95% CI: −4.97 to 0.64; *P* = 0.13), while omitting Liao et al. (32) resulted in MD = −2.55 s (95% CI: −7.30 to 2.20; *P* = 0.29), indicating model instability (Supplementary File 5). These findings highlight that the pooled result is highly sensitive to individual studies, and the conclusion should be interpreted with caution.

**Table 4:** Meta-analysis summary of differences in meibomian gland indicators between TAO and controls.

**Indicators**	**Number of studies**	**Eyes**	**Pooled mean difference**	**95%CI**	***P*-value**	***I* ^2^**
NITBUT-f (s)	5	587	−3.37	[−6.58, −0.16]	0.04^*^	95%
NITBUT-avg (s)	6	655	−2.05	[-4.34, 0.25]	0.08	91%
LLT (nm)	5	729	4.27	[1.11, 7.43]	0.008^**^	6%
Meiboscore	4	281	1.03	[0.68, 1.37]	< 0.001^***^	74%
Meibum quality	5	539	0.60	[−0.08, 1.29]	0.081	96%
TBUT (s)	6	501	−3.06	[−5.49, −0.63]	0.011^*^	98%
**MG dropout area**						
Upper eyelid (%)	3	364	11.38	[2.76, 20.00]	0.010^*^	94%
Lower eyelid (%)	2	152	9.08	[6.23, 11.93]	< 0.001^***^	0%

^*^ indicates *P* < 0.05, ^**^ indicates *P* < 0.01, ^***^ indicates *P* < 0.001; LLT lipid layer thickness, meiboscore, meibum quality, NITBUT-f first non-invasive tear film break-up time, NITBUT-avg average non-invasive tear film break-up time, TBUT tear break-up time, MAD meibomian gland acinar density, MALD meibomian gland acinar longest diameter, MASD meibomian gland acinar shortest diameter.

Six studies (31–33, 35, 36, 40) involving 655 eyes (Figure 2B and Table 4) demonstrated significantly shorter NITBUT-avg in patients with TAO compared to controls (MD = −2.05 s, 95% CI: −4.34 to 0.25; *P* = 0.08), with nonsignificant heterogeneity (*I*^2^ = 91%, *P* < 0.001). Subgroup analysis suggests that heterogeneity is not associated with device type. Sequential exclusion of individual studies did not alter the effect direction or statistical significance (*P* < 0.05), confirming the robustness of the conclusion (Supplementary File 5).

**Figure 3:**
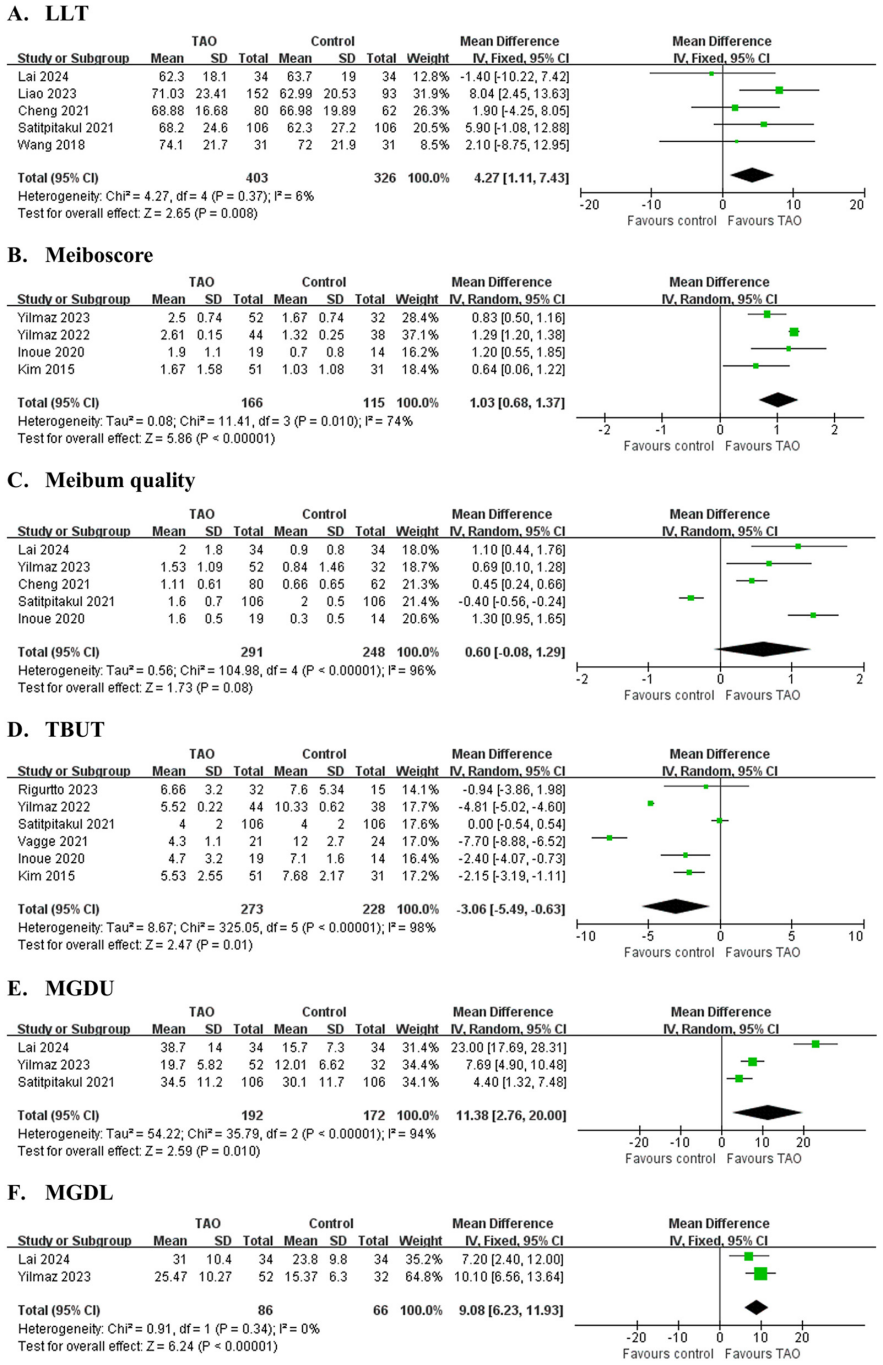
Forest plot of pooled difference in lipid layer thickness (LLT), meiboscore, meibum quality, tear break-up time (TBUT), meibomian glands dropout area of upper eyelid (MGDU) and lower eyelid (MGDL) between TAO and controls. **(A)** LLT; **(B)** Meiboscore; **(C)** Meibum quality; **(D)** TBUT; **(E)** MGDU; **(F)** MGDL.

Five studies (31–34, 42) involving 729 eyes (Figure 3A and Table 4) compared LLT between patients with TAO and controls. The pooled analysis showed significantly greater LLT in patients with TAO (MD = 4.27 nm, 95% CI: 1.11 to 7.4; *P* = 0.008), with no significant heterogeneity across studies (*I*^2^ = 6%, *P* = 0.37). However, sensitivity analysis revealed substantial dependence on the study by Liao et al. Exclusion of this study removed statistical significance (adjusted MD = 2.51 nm, 95% CI: −1.32 to 6.33; *P* = 0.20), indicating model instability (Supplementary File 5). These findings should be interpreted with caution due to the fragility of the pooled estimate.

Four studies (36, 37, 39, 44) involving 281 eyes (Figure 3B and Table 4) evaluated Meiboscore. Patients with TAO exhibited significantly higher Meiboscore than controls (MD = 1.03, 95% CI: 0.68 to 1.37; *P* < 0.001), although substantial heterogeneity was observed (*I*^2^ = 74%, *P* = 0.01). Sequential exclusion of individual studies did not alter the effect direction or statistical significance (*P* < 0.05), confirming the robustness of the conclusion.

Five studies (31, 33, 36, 42, 44) involving 539 eyes (Figure 3C and Table 4) found no significant difference in meibum quality between patients with TAO and controls (MD = 0.60, 95% CI: −0.08 to 1.29; *P* = 0.135), with high heterogeneity (I^2^ = 96%, P < 0.001). Sensitivity analysis revealed critical instability: exclusion of Satitpitakul et al. (42) shifted the MD to 0.87 (95% CI: 0.37 to 1.37; *P* < 0.001), reversing the statistical conclusion (Supplementary File 5). These findings suggest that the pooled result is highly sensitive to individual studies, and the conclusion should be interpreted with caution.

Six studies (37, 39, 40, 42–44) involving 501 eyes (Figure 3D and Table 4) found significantly shorter TBUT in patients with TAO compared to controls (MD = −3.06 s, 95% CI: −5.49 to −0.63; *P* = 0.011), with substantial heterogeneity (I^2^ = 98%, *P* < 0.001). Sensitivity analysis revealed model instability: exclusion of Yilmaz et al. (37) shifted the MD to −2.67 s (95% CI: −5.68 to 0.33; *P* = 0.08), while excluding Vagge et al. (43) resulted in MD = −2.10 s (95% CI:−4.80 to 0.59; *P* = 0.13), both eliminating statistical significance (Supplementary File 5). These findings highlight the fragility of the pooled estimate and underscore the need for cautious interpretation of the association between TAO and TBUT reduction.

Three studies (31, 36, 42) involving 364 eyes (Figure 3E and Table 4) demonstrated significantly greater meibomian gland dropout in the upper eyelid in patients with TAO compared to controls (MGDU: MD = 15.17%, 95% CI: 0.17 to 30.17; P = 0.046). However, sensitivity analysis revealed substantial dependence on the study by Yilmaz et al. (36) Exclusion of this study removed statistical significance (adjusted MD = 13.37%, 95% CI: −4.66 to 31.80; *P* = 0.14), indicating model instability (Supplementary File 5). These findings should be interpreted with caution due to the fragility of the pooled estimate. Two studies (31, 36) involving 152 eyes (Figure 3F and Table 4) demonstrated significantly greater meibomian gland dropout in the lower eyelid in patients with TAO compared to controls (MGDL: MD = 9.08%, 95% CI: 6.23 to 11.93; *P* < 0.001). Extreme heterogeneity was observed for MGDU (*I*^2^ = 96%, *P* < 0.001), while MGDL exhibited homogeneity (*I*^2^ = 0%, *P* = 0.34).

Two studies incorporated three IVCM (*in vivo* confocal microscopy) indicators: MAD, MASD, and MALD. Cheng et al. (33) reported statistically significant differences between TAO and controls across all three indicators: MAD (83.5 ± 34.3 vs 114.7 ± 34.9, *P* < 0.001), MALD (119.5 ± 28.6 vs. 58.7 ± 20.3, *P* < 0.001), and MASD (45.5 ± 19.9 vs 27.8 ± 9.9, *P* < 0.001). Similarly, Vagge et al. (43) demonstrated significant differences in MAD (24.5 ± 9.1 vs. 34.2 ± 7.5, *P* < 0.001), MALD (94.4 ± 21.2 vs. 64.3 ± 10.1, *P* < 0.001), and MASD (56.6 ± 15.3 vs. 42.2 ± 12.3, *P* = 0.001) between TAO and controls. Methodological variations existed between studies. Vagge et al. (43) measured MAD explicitly at 20–70 μm below the epithelial surface, whereas Cheng et al. provided no imaging depth specification. This discrepancy may introduce measurement variability. Furthermore, the limited number of available studies potentially affects statistical stability. Thus, this study did not pool IVCM data from the two studies for meta-analysis.

#### 3.4.2 Meibomian gland indicators of patients with active TAO vs. inactive TAO

**Figure 4:**
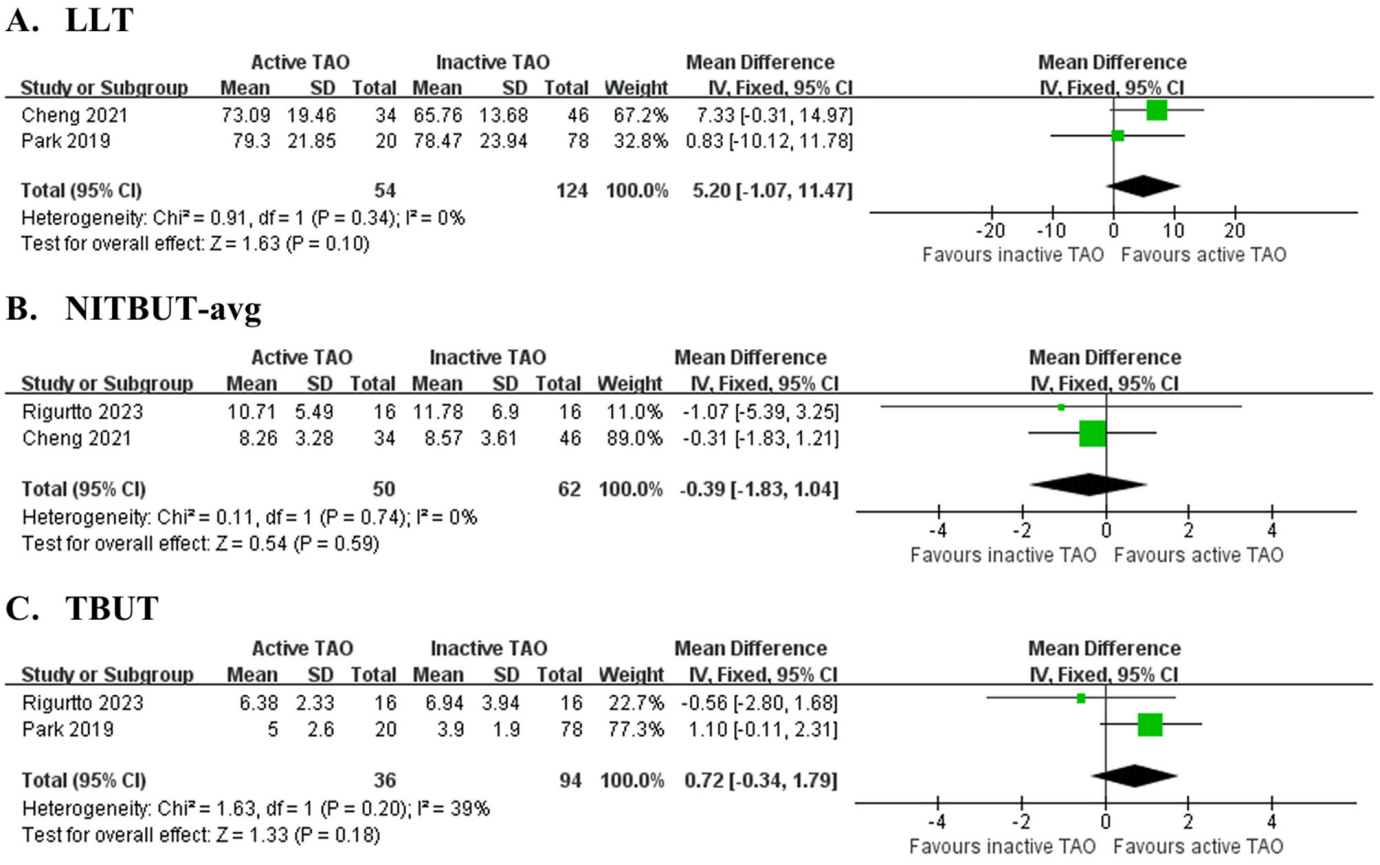
Forest plot of pooled difference in lipid layer thickness (LLT), average non-invasive tear film break-up time (NITBUT-avg) and tear break-up time (TBUT) between active TAO and inactive TAO. **(A)** LLT,; **(B)** NITBUT-avg; **(C)** TBUT.

Two studies (33, 38) involving 178 eyes (Figure 4A and Table 5) compared LLT between active and inactive TAO, finding no significant difference (MD = 5.20 s, 95% CI: −1.07 to 11.47; *P* = 0.101), with no heterogeneity observed between studies (*I*^2^ = 0%, *P* = 0.34). Two studies (33, 40) involving 112 eyes (Figure 4B and Table 5) compared NITBUT avg between active and inactive TAO, finding no significant difference (MD = −0.39 s, 95% CI: −1.83 to 1.04; *P* = 0.594), with no heterogeneity observed between studies (*I*^2^ = 0%, *P* = 0.74). Two studies (38, 40) involving 130 eyes (Figure 4C and Table 5) compared TBUT between active and inactive TAO, showing no significant difference (MD = 0.72 s, 95% CI: −0.34 to 1.79; *P* = 0.183), with nonsignificant heterogeneity (*I*^2^ = 39%, *P* = 0.020).

**Table 5:** Meta-analysis summary of differences in meibomian gland indicators between active TAO and inactive TAO.

**Indicators**	**Number of studies**	**Eyes**	**Pooled mean difference**	**95%CI**	***P*-value**	***I* ^2^**
LLT (nm)	2	178	5.20	[−1.07, 11.47]	0.101	0%
NITBUT-avg (s)	2	112	−0.39	[−1.83, 1.04]	0.594	0%
TBUT (s)	2	130	0.72	[−0.34, 1.79]	0.183	39%

^*^indicates *P* < 0.05, ^**^ indicates *P* < 0.01, ^***^indicates *P* < 0.001; LLT lipid layer thickness, NITBUT-avg average non-invasive tear film break-up time, TBUT tear break-up time.

## 4 Discussion

This meta-analysis systematically evaluated meibomian gland characteristics in TAO, incorporating 13 studies with 813 TAO eyes and 522 healthy control eyes. The results reveal significant structural and functional abnormalities in patients with TAO compared to controls. Structurally, TAO eyes exhibit more severe gland dropout, higher Meiboscores and higher LLT. Functionally, patients with TAO show reduced tear film stability, evidenced by shorter NITBUT-f and TBUT values. Collectively, these findings suggest that TAO accelerates MGD through a distinct pathophysiology, leading to irreversible structural damage and potential compensatory responses.

Structural changes in the meibomian glands are a key feature of TAO, including increased gland dropout and abnormal morphology. Our results demonstrate higher Meiboscores and larger gland loss areas in patients with TAO, providing direct evidence of gland atrophy and dropout (45). Notably, superior eyelid gland loss is more pronounced than inferior eyelid loss, suggesting that upper eyelids are more severely affected. This may be due to their larger exposed surface area, which increases exposure to inflammatory factors. Hwang et al. (46) proposed that ocular surface inflammation in TAO directly correlates with meibomian gland structural damage. The larger exposed area of the upper eyelids likely increases inflammatory exposure, explaining their greater involvement. Additionally, Luo et al. (47) found that orbital fat and muscle expansion in TAO mechanically compresses the eyelids, deforming meibomian ducts and obstructing lipid secretion. *In vivo* confocal microscopy revealed no significant difference in acinar density between patients with TAO and controls, but patients with TAO exhibited significantly enlarged acinar diameters. This may indicate compensatory hypertrophy of remaining acini after adjacent gland dropout or gland dilation caused by inflammation-induced lipid retention (33).

Meibomian gland structural damage directly impairs tear film stability. Our meta-analysis reveals an uncertain difference in the NIBUT-f between TAO patients and controls: while the pooled results showed a trend of shortening, sensitivity analysis indicated high dependence on individual studies. In contrast, the observed shortening of NITBUT-avg demonstrated greater consistency, with sensitivity analyses indicating relative stability in pooled estimates. The observed discrepancy could be partially attributed to the properties of these metrics: NITBUT-avg, by averaging multiple measurements, tends to mitigate random measurement fluctuations, whereas NITBUT-f appears more susceptible to transient perturbations such as pre-measurement blink patterns or short-term ocular surface microenvironmental changes. Furthermore, the subjective TBUT indicator also demonstrated fragility in sensitivity analysis. This may be potentially linked to methodological factors such as fluorescein interference or assessment variability, which could affect the robustness of the findings. These findings collectively suggest that impaired gland structure accelerates tear evaporation by disrupting tear film lipid layer stability (48). Mantelli et al. (49) suggested that inflammation leads to excessive tear evaporation by reducing membrane-associated mucins in the cornea and conjunctiva, as well as secretory mucins from goblet cells. Tear film stability depends not only on the lipid layer but also on the mucin layer secreted by conjunctival goblet cells. The rapid tear breakup observed in patients with TAO is likely due to defects in both layers. Notably, this study found no significant differences in NITBUT-avg or TBUT between active and inactive TAO, suggesting that tear film stability may not be influenced by disease activity. However, the limited sample size warrants further investigation to validate these findings.

LLT in patients with TAO exhibits complex patterns. Our meta-analysis demonstrated significantly greater LLT in TAO patients compared to controls. Our study found increased, rather than decreased, LLT in patients with TAO compared to controls. Although periglandular inflammation contributes to MGD, compensatory secretion from residual glands and forceful blinking due to lagophthalmos may lead to increased lipid deposition on the tear film (50, 51). Finis et al. (52) noted that meibomian glands can maintain baseline function even with over 40% gland dropout. In active TAO, significant proptosis increases eyelid tension and blink force, forcibly expelling meibum and enhancing LLT (53). Paradoxically, our study demonstrated no significant difference in LLT between active and inactive TAO groups. Additionally, our analysis showed no significant difference in meibum quality between patients with TAO and controls. While substantial meibomian gland loss in TAO would typically reduce meibum quality, Farid et al. suggest that ocular surface inflammation may alter secretion quality. However, Iqbal and Shakya (54, 55) propose that lipid metabolism in TAO-associated MGD may differ from conventional MGD. Additionally, the current meibum quality assessment may overlook subtle compositional changes in TAO, warranting molecular-level studies to confirm this hypothesis.

TAO-associated MGD involves anatomical, inflammatory, and molecular mechanisms. Anatomically, mechanical compression from orbital tissue expansion and impaired blinking due to eyelid retraction obstruct meibum excretion. Inflammatory processes include tear-mediated autoimmune reactions, where elevated pro-inflammatory cytokines drive meibomian gland fibrosis and atrophy. Notably, increased corneal Langerhans cell density distinguishes TAO from conventional MGD (56–58). Molecular studies indicate that IGF-1R signaling—central to TAO pathology—induces meibomian epithelial apoptosis via the PI3K/Akt pathway. Concurrently, oxidative stress-induced reactive oxygen species may activate the NLRP3 inflammasome, further exacerbating gland damage (59, 60). This multifactorial pathology underpins the complex and refractory nature of TAO-associated MGD.

Our findings may offer valuable clinical guidance for managing TAO. Given the high prevalence and severity of MGD in patients with TAO, conventional dry eye treatments often provide limited benefits (61). Routine comprehensive assessments should be adopted, incorporating meibomian gland imaging, meibum quality evaluation, and tear film stability measurements. Early detection and intervention for MGD, even in subclinical stages, could help delay progressive gland damage. For treatment, this study recommends combining systemic immunosuppression with specific therapies, such as thermal pulsation and intense pulsed light treatments (62, 63). This integrated approach enhances meibum excretion while controlling local inflammation, as supported by existing studies.

This study has several limitations. First, the limited number of included studies and generally small sample sizes may have reduced statistical power, making it difficult to detect subtle differences in certain indicators, which constrained the reliability of pooled estimates.

Second, Methodological differences may have introduced potential heterogeneity. Specifically, different devices such as the Sirius and OCULUS Keratograph 5M were used to measure NITBUT. Variations in measurement principles and algorithms may have compromised the comparability of results. The assessment of meibomian gland dropout also lacked standardization: some studies used manual quantification with ImageJ software, while others relied on automated analysis with the Sirius system. This discrepancy between manual and automated methods could introduce inter-observer bias and systematic error. Furthermore, meibomian gland imaging protocols were not uniform; only Vagge et al. (43) explicitly specified the imaging depth. For subjective indicators such as meibum quality and TBUT, although certain criteria were applied, inter-observer variation in assessment remains a possibility (64).

Third, there were shortcomings in patient selection and grouping. Only four studies explicitly excluded TAO patients with a pre-existing DED diagnosis or prior DED treatment, while the remaining studies did not specify such exclusions, potentially introducing population heterogeneity. In the control groups, seven studies excluded individuals with a history of DED, whereas others simply described controls as “healthy” without verification, which may have led to selection bias. Moreover, due to the predominance of cross-sectional or case-control designs, the temporal relationship between DED and TAO onset could not be determined. Variations in TAO disease duration and severity among patients may influence ocular surface and meibomian gland status (65). Future cohort studies should analyze ocular surface or meibomian gland function indicators in patients with and without DED, both before and after TAO diagnosis.

Fourth, there exists a lack of standardization in defining disease activity. Some included studies failed to categorize TAO cases into active or inactive groups based on the CAS. Furthermore, even studies analyzing active subgroups did not report specific CAS scores for each cohort. This variability in inflammation levels could confound interpretations of meibomian gland dysfunction outcomes, potentially introducing bias in result interpretation.

Finally, the inclusion of multi-regional and multi-ethnic data may introduce heterogeneity due to racial differences in eyelid anatomy and MGD epidemiology (66, 67). Future high-quality, large-scale studies are needed to validate these findings and enhance statistical power. Additionally, newly published clinical studies may be incorporated into future meta-analyses to strengthen the conclusions.

In conclusion, this meta-analysis provides systematic quantitative evidence that MGD is a common ocular manifestation in TAO. Patients with TAO exhibit significant structural damage and functional decline in meibomian glands, with a pathology characterized by chronic, progressive, and potentially irreversible changes. These findings highlight the need for clinical practice to adopt targeted MGD assessment and long-term management strategies. More specific interventions are required to effectively improve patients' long-term visual function and quality of life.

## Data Availability

The original contributions presented in the study are included in the article/Supplementary material, further inquiries can be directed to the corresponding author.
