# Supplementary material for: Meibomian gland dysfunction in patients with thyroid-associated ophthalmopathy: a systematic review and meta-analysis
Source: Front Med (Lausanne). 2025 Nov 11;12:1709057. doi: 10.3389/fmed.2025.1709057 (PMC12643990; doi:10.3389/fmed.2025.1709057)
Supplement: Supplementary file 3 [file Supplementary_file_3.docx]

**Supplementary File 3**

Table 1 Systematic search strategy formulations of each database

| **PubMed** From inception to March 5th, 2025 | | |
| --- | --- | --- |
| #1 | ("meibomian glands"[MeSH Terms] OR "meibomian gland dysfunction"[MeSH Terms] OR "MGD"[Title/Abstract] OR "meibomian gland dysfunction"[Title/Abstract] OR "meibomian glands"[Title/Abstract]) AND ("graves ophthalmopathy"[MeSH Terms] OR "graves ophthalmopathy"[Title/Abstract] OR "thyroid eye disease"[Title/Abstract] OR "thyroid associated ophthalmopathy"[Title/Abstract] OR "thyroid associated orbitopathy"[Title/Abstract] OR "graves orbitopathy"[Title/Abstract] OR "graves eye disease"[Title/Abstract]) | **30** |
| **Scopus** From inception to March 5th, 2025 | | |
| #1 | (TITLE-ABS-KEY(graves ophthalmopathy) OR TITLE-ABS-KEY(thyroid eye disease) OR TITLE-ABS-KEY(thyroid associated orbitopathy) OR TITLE-ABS-KEY(thyroid associated ophthalmopathy) OR TITLE-ABS-KEY(graves orbitopathy) OR TITLE-ABS-KEY(graves eye disease)) AND (TITLE-ABS-KEY(meibomian glands) OR TITLE-ABS-KEY(meibomian gland dysfunction) OR TITLE-ABS-KEY(MGD)) | **64** |
| **Web of science** All From inception to March 5th, 2025 | | |
| #1 | ((((((((((((AB=(graves ophthalmopathy)) OR TI=(graves ophthalmopathy)) OR TI=(thyroid eye disease)) OR AB=(thyroid eye disease)) OR AB=(thyroid associated ophthalmopathy)) OR TI=(thyroid associated ophthalmopathy)) OR AB=(thyroid associated orbitopathy)) OR TI=(thyroid associated orbitopathy)) OR AB=(graves orbitopathy)) OR TI=(graves orbitopathy)) OR AB=(graves eye disease)) OR TI=(graves eye disease)) | **—** |
| #2 | ((((((AB=(meibomian glands)) OR TI=(meibomian glands)) OR TI=(meibomian gland dysfunction)) OR AB=(meibomian gland dysfunction)) OR AB=(MGD)) OR TI=(MGD)) | **—** |
| #3 | #1 AND #2 | **28** |
| **Cochrane** From inception to March 5th, 2025 | | |
| #1 | MeSH descriptor: [Graves Ophthalmopathy] explode all trees | **201** |
| #2 | (graves ophthalmopathy):ti,ab,kw | **434** |
| #3 | (thyroid eye disease):ti,ab,kw | **370** |
| #4 | (thyroid associated ophthalmopathy):ti,ab,kw | **136** |
| #5 | (thyroid associated orbitopathy):ti,ab,kw | **31** |
| #6 | (graves orbitopathy):ti,ab,kw | **153** |
| #7 | (graves eye disease):ti,ab,kw | **263** |
| #8 | #1 or #2 or #3 or #4 or #5 or #6 or #7 | **667** |
| #9 | MeSH descriptor: [Meibomian Glands] explode all trees | **157** |
| #10 | MeSH descriptor: [Meibomian Gland Dysfunction] explode all trees | **134** |
| #11 | (meibomian glands):ti,ab,kw | **309** |
| #12 | (meibomian gland dysfunction):ti,ab,kw | **555** |
| #13 | (MGD):ti,ab,kw | **10999** |
| #14 | #9 or #10 or #11 or #12 or #13 | **11294** |
| #15 | #8 and #13 | **8** |
| **Embase** From inception to March 5th, 2025 | | |
| #1 | 'meibomian gland dysfunction'/exp | **2283** |
| #2 | 'meibomian gland'/exp | **3334** |
| #3 | 'meibomian gland dysfunction':ab,ti | **2043** |
| #4 | 'meibomian glands':ab,ti | **1540** |
| #5 | 'MGD':ab,ti | **3147** |
| #6 | #1 OR #2 OR #3 OR #4 OR #5 | **7078** |
| #7 | 'endocrine ophthalmopathy '/exp | **7814** |
| #8 | 'graves ophthalmopathy':ab,ti | **2487** |
| #9 | 'thyroid eye disease':ab,ti | **2002** |
| #10 | 'thyroid associated orbitopathy':ab,ti | **379** |
| #11 | 'thyroid associated ophthalmopathy':ab,ti | **1185** |
| #12 | 'graves orbitopathy':ab,ti | **1723** |
| #13 | 'graves eye disease':ab,ti | **57** |
| #14 | #7 OR #8 OR #9 OR #10 OR #11 OR #12 OR#13 | **9454** |
| #15 | #6 AND #14 | **44** |
| **Medline** From inception to March 5th, 2025 | | |
| #1 | (MH "Graves Ophthalmopathy") | **—** |
| #2 | Tl graves ophthalmopathy OR AB graves ophthalmopathy OR Tl thyroid eye disease OR AB thyroid eye disease OR Tl thyroid associated ophthalmopathy OR AB thyroid associated ophthalmopathy OR Tl thyroid associated orbitopathy OR AB thyroid associated orbitopathy OR Tl graves orbitopathy OR AB graves orbitopathy OR Tl graves eye disease OR AB graves eye disease | **5943** |
| #3 | (MH "Meibomian gland dysfunction") OR (MH "Meibomian glands ") | **—** |
| #4 | Tl Meibomian Gland Dysfunction OR AB Meibomian Gland Dysfunction ORTI Meibomian Glands OR AB Meibomian Glands OR TI MGD OR AB MGD | **4290** |
| #5 | S1 OR S2 | **4623** |
| #6 | S3 OR S4 | **6444** |
| #7 | S5 AND S6 | **26** |
