# Supplementary material for: Meibomian gland dysfunction in patients with thyroid-associated ophthalmopathy: a systematic review and meta-analysis
Source: Front Med (Lausanne). 2025 Nov 11;12:1709057. doi: 10.3389/fmed.2025.1709057 (PMC12643990; doi:10.3389/fmed.2025.1709057)
Supplement: Supplementary file 4 [file Supplementary_file_4.docx]

**Supplementary File 4**

**
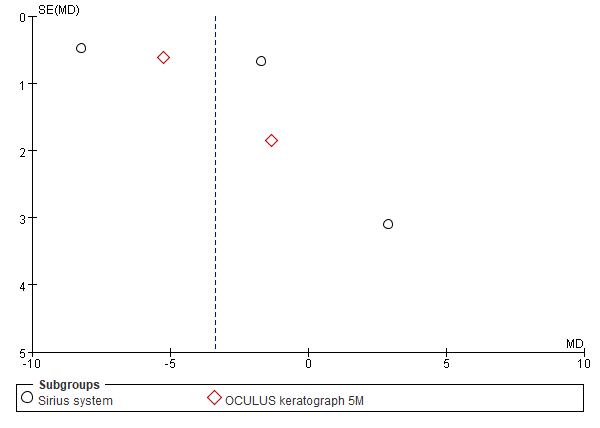
**Figure 1: Funnel plot in first non-invasive tear film break-up time (NITBUT-f) between TAO and controls.

**
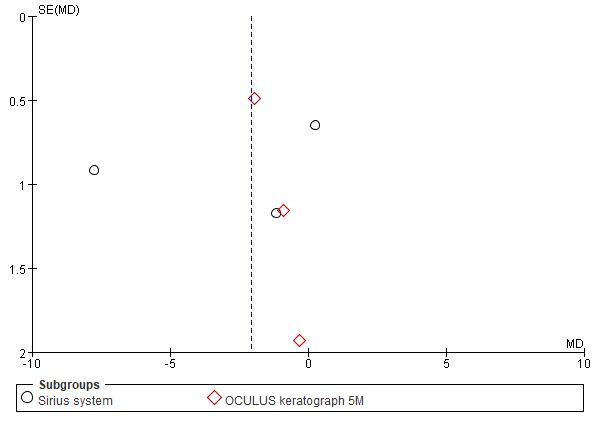
**Figure 2: Funnel plot in average non-invasive tear film break-up time (NITBUT-avg) between TAO and controls.


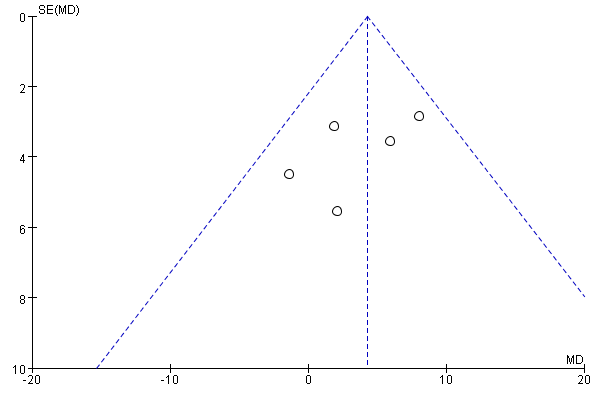


Figure 2: Funnel plot in lipid layer thickness (LLT) between TAO and controls.


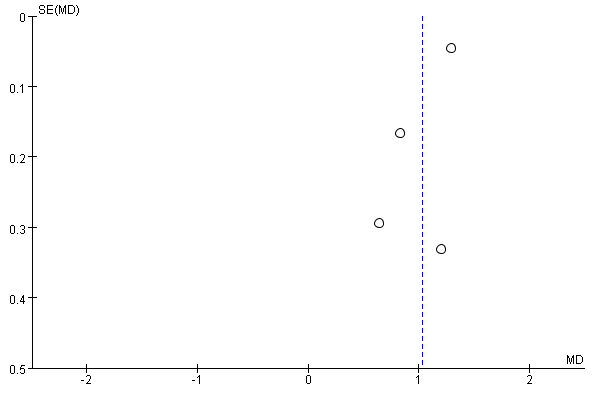


Figure 3: Funnel plot in meiboscore between TAO and controls.


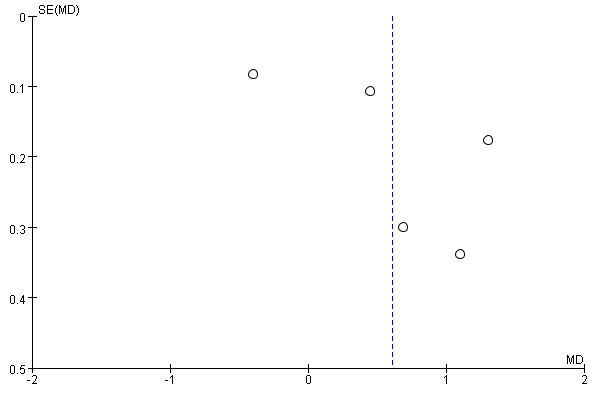


Figure 5: Funnel plot in meibum quality between TAO and controls.


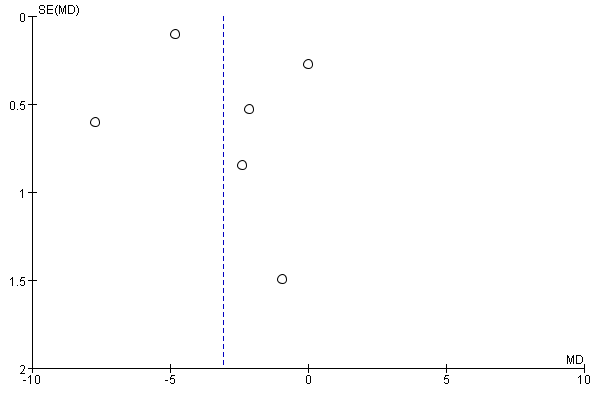
 Figure 6: Funnel plot in tear break-up time (TBUT) between TAO and controls.


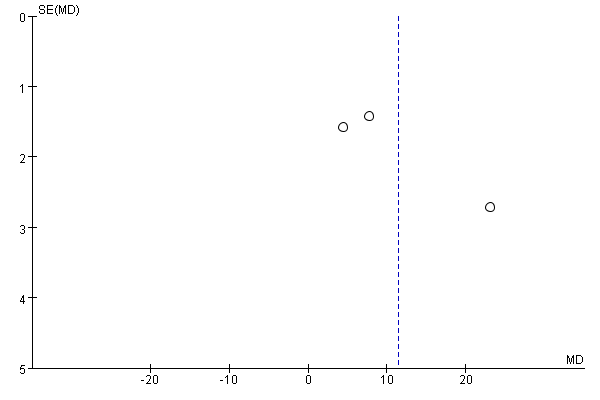
Figure 7: Funnel plot in meibomian glands dropout area of upper eyelid (MGDU) between TAO and controls.


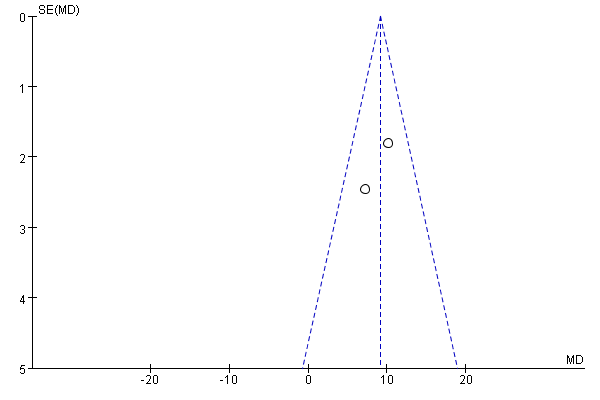
 Figure 8: Funnel plot in meibomian glands dropout area of lower eyelid (MGDL) between TAO and controls.


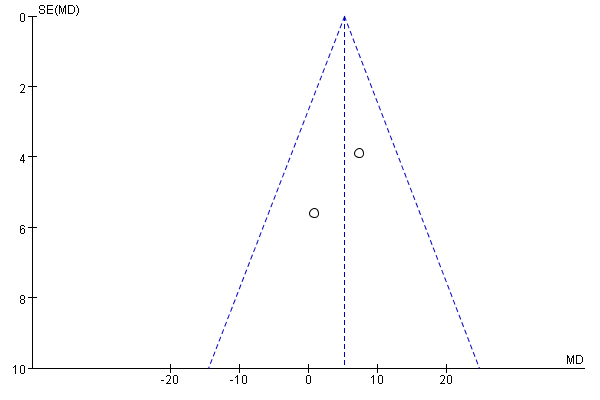
Figure 9: Funnel plot in lipid layer thickness (LLT) between active TAO and inactive TAO.


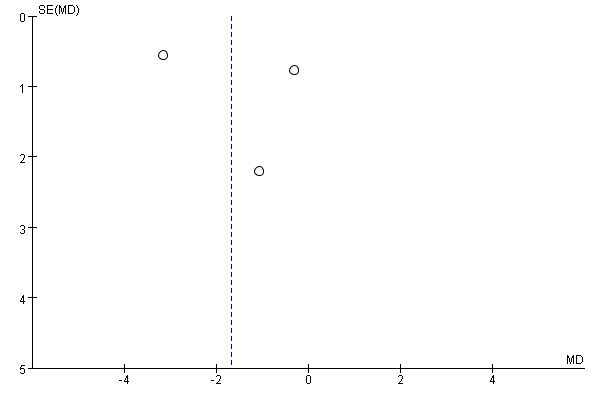
 Figure 10: Funnel plot in average non-invasive tear film break-up time (NITBUT-avg) between active TAO and inactive TAO.


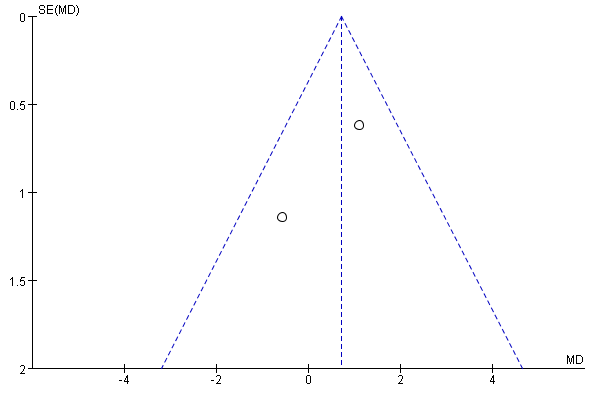
 Figure 11: Funnel plot in tear break-up time (TBUT) between active TAO and inactive TAO.
