# Supplementary material for: Meibomian gland dysfunction in patients with thyroid-associated ophthalmopathy: a systematic review and meta-analysis
Source: Front Med (Lausanne). 2025 Nov 11;12:1709057. doi: 10.3389/fmed.2025.1709057 (PMC12643990; doi:10.3389/fmed.2025.1709057)
Supplement: Supplementary file 5 [file Supplementary_file_5.docx]

**Supplementary File 5**
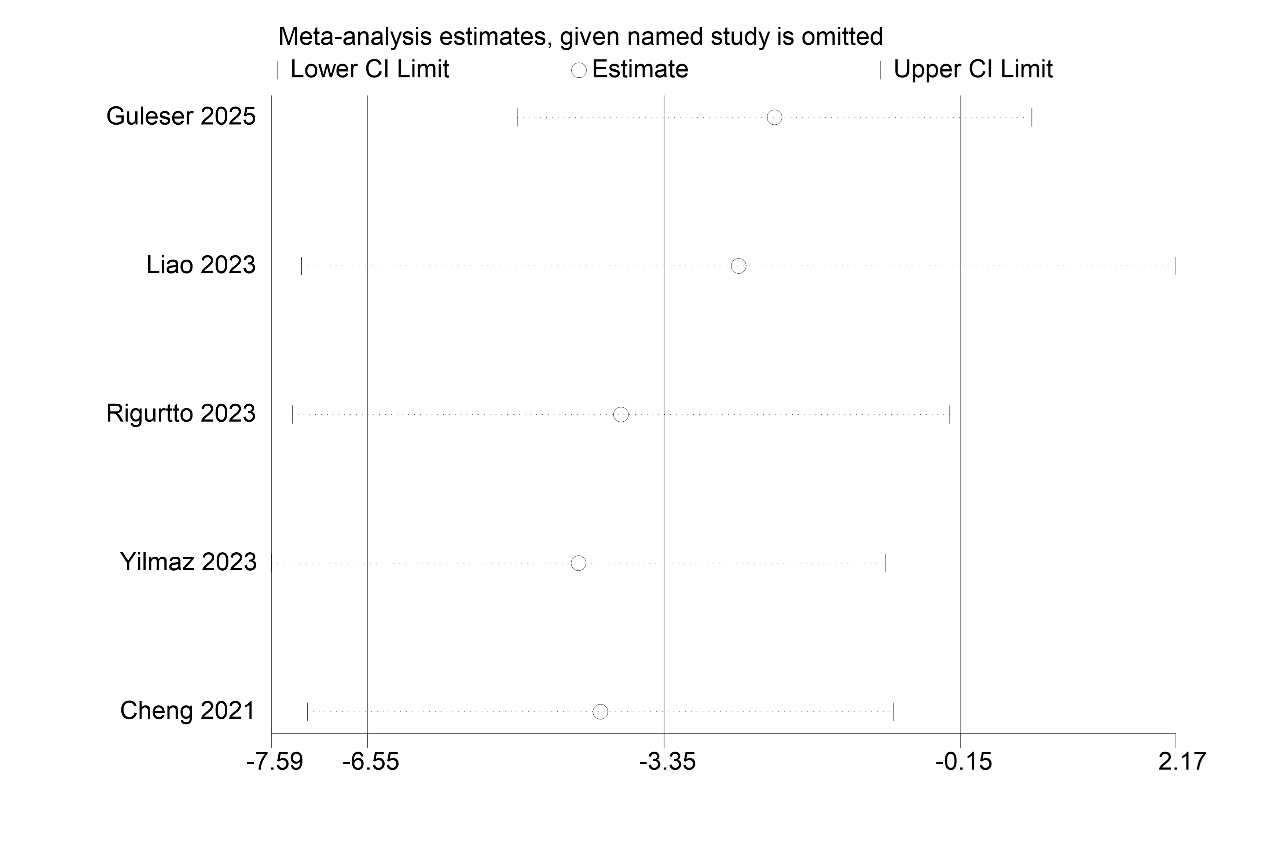
 Figure 1: Sensitivity analysis in first non-invasive tear film break-up time (NITBUT-f) between TAO and controls.


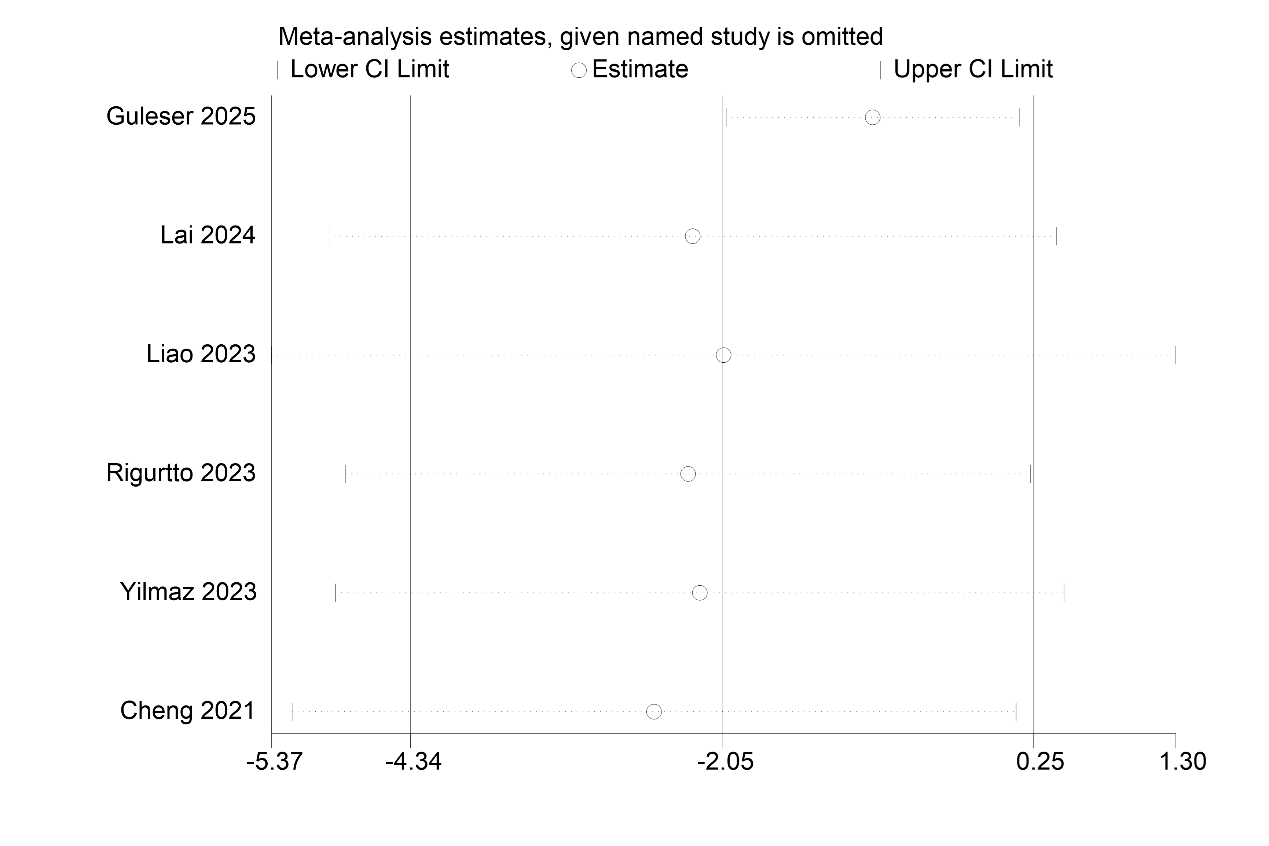
Figure 2: Sensitivity analysis in average non-invasive tear film break-up time (NITBUT-avg) between TAO and controls.


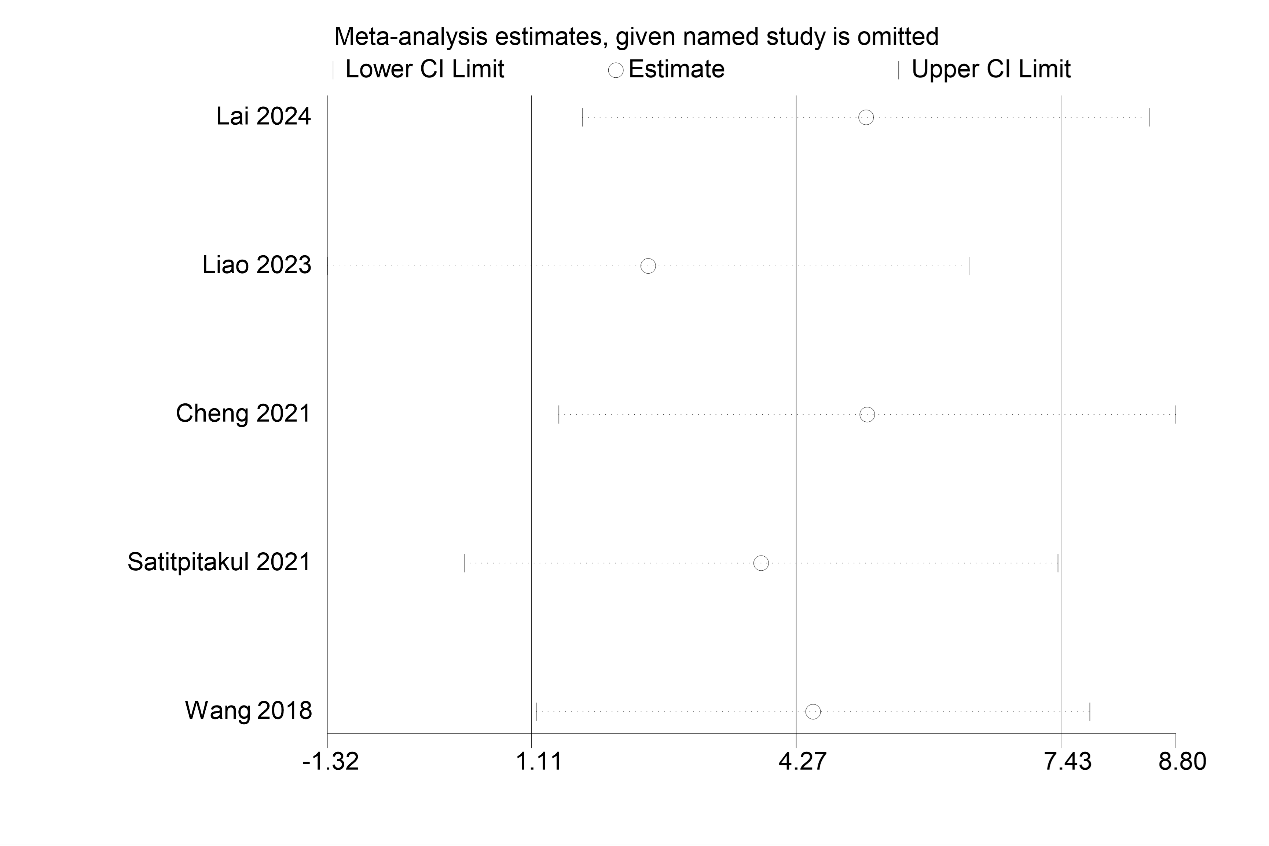
 Figure 3: Sensitivity analysis in lipid layer thickness (LLT) between TAO and controls.


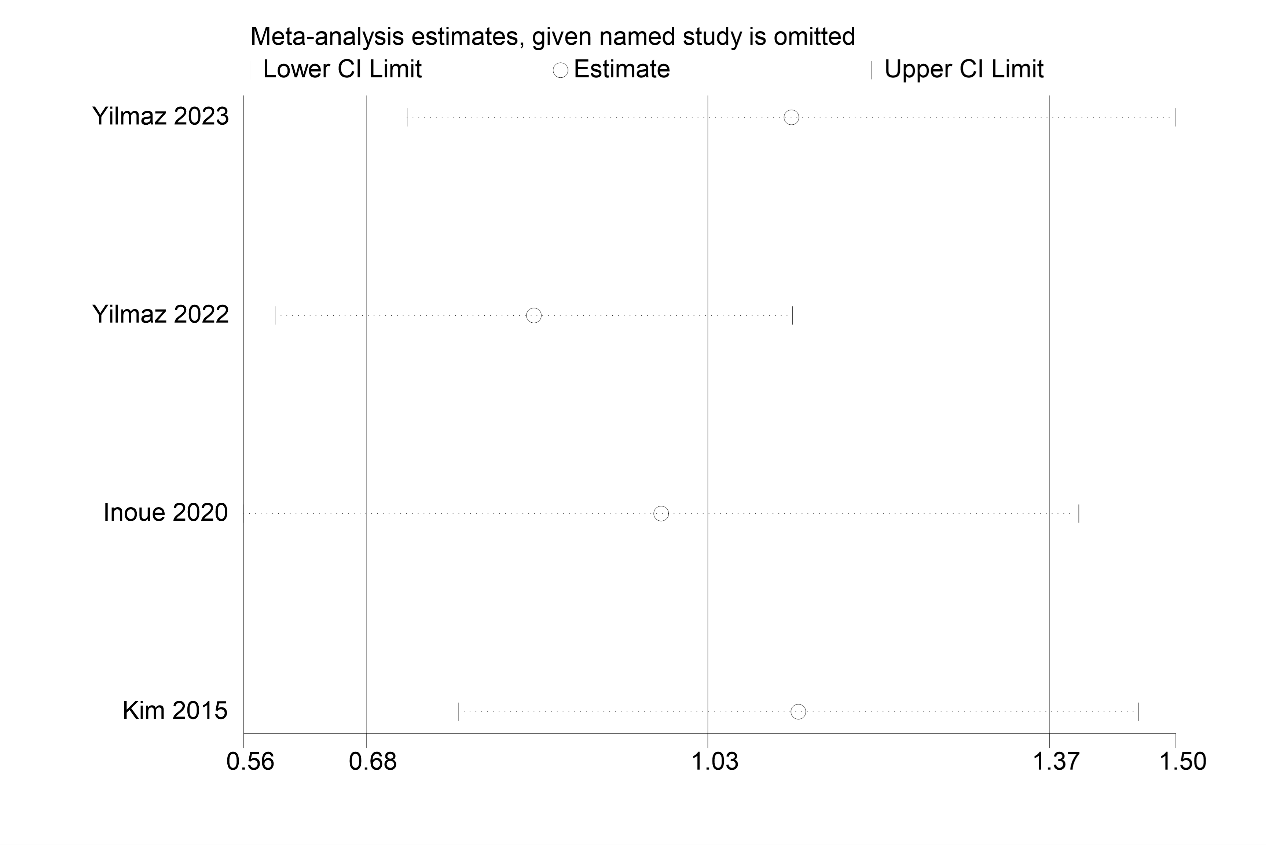
Figure 4: Sensitivity analysis in Meiboscore between TAO and controls.


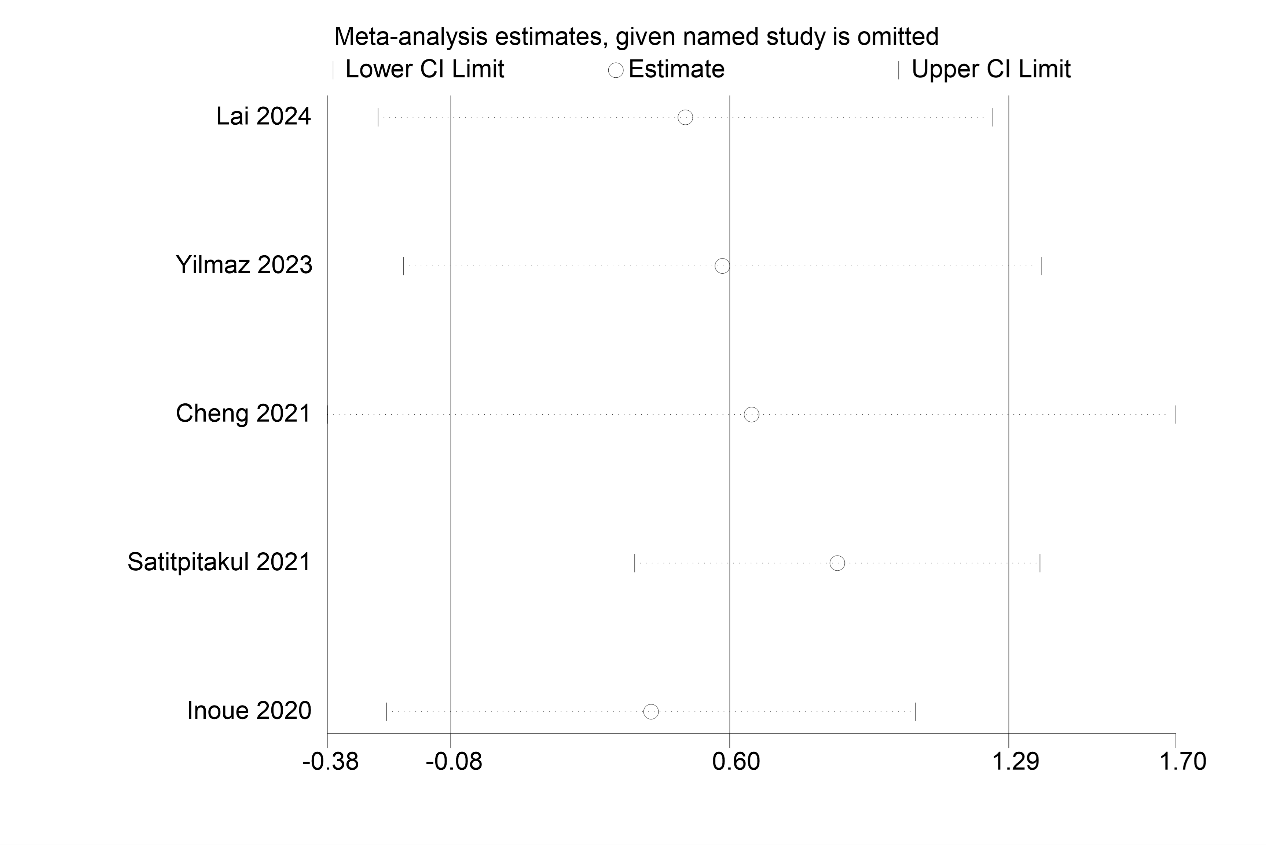


Figure 5: Sensitivity analysis in Meibum quality between TAO and controls.


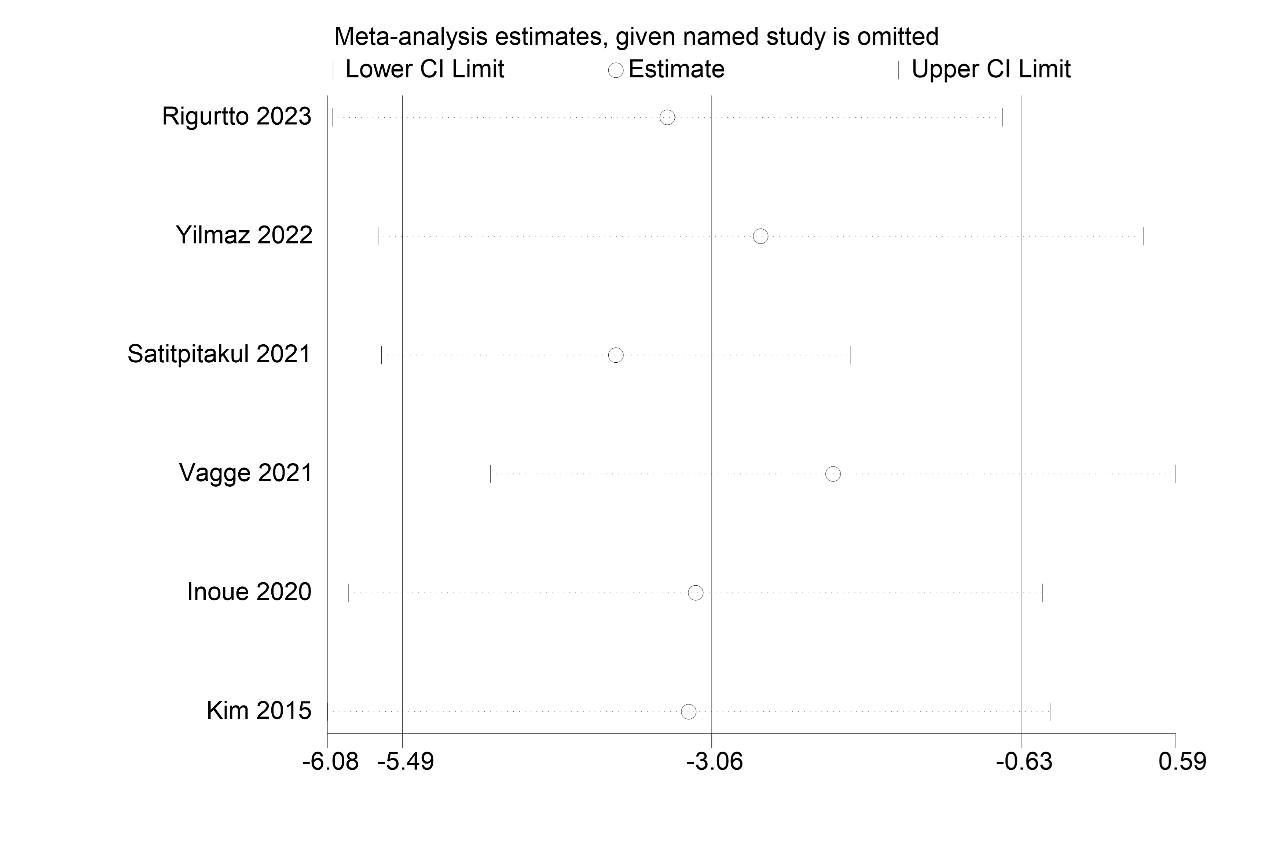


Figure 6: Sensitivity analysis in tear break-up time (TBUT) between TAO and controls

 Figure 7: Sensitivity analysis in meibomian glands dropout area of upper eyelid (MGDU) between TAO and controls
